# Contrasting phagocyte respiratory burst responses in two European bat species during hibernation

**DOI:** 10.3389/fimmu.2026.1826565

**Published:** 2026-05-19

**Authors:** Jan Zukal, Monika Nemcova, Erik Bachorec, Tomas Heger, Veronika Seidlova, Ivana Papezikova, Vladimir Piacek, Katerina Zukalova, Jiri Pikula

**Affiliations:** 1 Department of Botany and Zoology, Masaryk University, Brno, Czechia; 2 Institute of Vertebrate Biology, Czech Academy of Sciences, Brno, Czechia; 3 Department of Ecology and Diseases of Zoo Animals, Game, Fish and Bees, University of Veterinary Sciences Brno, Brno, Czechia

**Keywords:** chiroptera, eco-immunology, innate immunity, phagocytosis, torpor

## Abstract

Bats use various natural or artificial structures as hibernacula, resulting in a diversification of hibernation strategies. For example, caves provide a stable thermal microclimate for the greater mouse-eared bat (*Myotis myotis*), whereas above-ground roosts used by the common noctule bat (*Nyctalus noctula*) provide much less protection from external weather conditions. These different ecological requirements result in distinct torpor-arousal patterns and movement activity, which significantly affect trade-offs between investing energy in immune functions and responses versus other biological functions. To examine potential functional differences during hibernation, we compared phagocyte respiratory burst in the two bat species. Our results show that *N. noctula* bats exhibit stronger and faster phagocytic immune responses than *M. myotis* bats. These results suggest that distinct hibernation strategies might reflect functional differences necessary for species-specific needs during hibernation and provide a foundation for future comparative studies across a broader range of bat species.

## Introduction

Seasonality at high latitudes is primarily associated with significant changes in temperature and food availability. The dramatic decline of temperature and food availability during winter poses a major challenge for many endotherms, which have had to evolve various adaptations to survive. Some species migrate to regions with more favourable conditions, while others have evolved heterothermy, which utilizes torpor to minimize metabolic needs (1). Most European bats belong to the second group and thus must balance the physiological costs of deep torpor, e.g. water loss, accumulation of metabolic waste, sleep deprivation and/or immune system inhibition (2–5). While our knowledge of the impact of hibernation on the immune system remains limited, some studies have demonstrated that lymphocyte activation, complement factors activity, antibody production and the acute phase response to lipopolysaccharide are all decreased during torpor (6–11). Hibernators also have a lower number of circulating leukocytes and neutrophils in peripheral blood (12). As reduced immune system function can have fatal consequences, bats have adapted at organismal, organ, cellular or molecular levels (13, 14), with most of these functions being quickly reactivated upon arousal (4, 15).

European bats begin hibernating in early October, while final arousals occur in mid-April, giving a total hibernation season length of nearly 200 days (16–18). However, not all individuals or populations follow the same hibernation model (19–22). Undoubtedly, the stable environment of underground hibernacula provides the conditions necessary for successful survival of hibernation. Nevertheless, even here, the bat hibernation season can be divided into three distinct periods, i.e. early, deep and late hibernation (23, 24). The continuous arrival of bats at the hibernaculum ends in mid‐December, after which movement activity starts again in mid‐March, with bats gradually departing from the shelter. The three-month deep hibernation period is characterized by low movement activity in the hibernaculum and minimal changes in bat numbers (25, 26). In contrast, above-ground hibernators are exposed to much more variable microclimate conditions. In such shelters, they can easily monitor ambient temperature and sense favourable foraging conditions (27). Consequently, these hibernators exhibit much more frequent movements than in cave-hibernating species (28, 29), though winter flight activity remains ubiquitous among temperate zone bats (30). Phagocytosis, a protective mechanism that links innate and adaptive immune responses (31), differs between homoiotherms and heterotherms (11). Nevertheless, two heterothermic bat species have been shown to display comparable time-related parameters of phagocyte activity, albeit with distinct quantitative values (11). As hibernation is an adaptation primarily designed to conserve energy, and the immune system is costly in terms of energetic expenditure, we would expect hibernators to reduce their immune response during torpor, only reactivating it during periodic arousals (8). When studying hibernating temperate bat species, it is important to understand the seasonal dynamics associated with the immune response.

To this end, we conducted a comparative study of cave-hibernating greater mouse-eared bats (*Myotis myotis*) and above-ground hibernating common noctule bats (*Nyctalus noctula*), regularly sampling the bats during deep hibernation period to describe changes in the phagocyte respiratory burst. While differences in torpor expression and winter movement activity during deep hibernation period may affect immune function and response, we acknowledge that the study species are also phylogenetically and ecologically distinct, and that these factors are intertwined. Thus, variability in immune response might arise from their combined influence. Within this framework, we predicted 1) a decrease in the phagocyte respiratory burst during deep hibernation in both hibernators, with different magnitudes related to species-specific hibernation behaviour and movement activity; and 2) individual differences related to the asynchrony of torpor/arousal bouts in the hibernating bats.

## Materials and methods

A total of 43 *M. myotis* and 43 N*. noctula* specimens were sampled during the winter of 2017 and 2018. Wild *M. myotis* bats were collected at the Sloupsko-Šošůvské caves [Czech Republic; 49.4104556 N, 16.7390147 E], while *N. noctula* bats were rescued from a factory building in Ivanovice na Hané [Czech Republic; 49.3054183 N, 17.0934306E]. Afterwards, individuals of both species were taken to accredited artificial hibernaculum at wildlife rescue centre at the Department of Ecology and Diseases of Zoo Animals, Game, Fish and Bees (University of Veterinary Sciences Brno, Czech Republic), where the sampling of both species was conducted. For the remainder of the hibernation period, bats of both species were kept in the artificial hibernaculum at 8 °C. During April and May, they were kept in a box at room temperature for ca. 1.5 months and they were fed mealworms and had unlimited access to water during this period. The number of specimens of both species sampled on each occasion was as follow: December 2017 (n = 10 and 11, respectively), January (n = 10 and 9), March (n = 12 and 11), and May 2018 (n = 11 and 12), with the first three samplings covering the deep hibernation period, while the last represented the active season. Before blood sampling, the bats were gently removed from the hibernaculum wall or box and placed in a cotton bag for 30 minutes to allow them to rewarm from their heterothermic state. The uropatagium was disinfected with alcohol, after which the uropatagial vessel was punctured using a sterile needle (23 G) and a 120 μl blood samples (approx. 0.6% of bat body mass) collected into a prepared tube using a heparinised pipette tip (20, 32). The puncture site was then compressed and sealed with a drop of surgical tissue glue (Surgibond, SMI AG, Belgium) to stop bleeding. Prior to release, the bats were given a subcutaneous dose of Ringer's lactate solution and 5% glucose to replace energy and fluids. All bats were released at the capture site and/or returned to an artificial hibernaculum within one hour of capture for blood sampling. No animal was sampled twice.

Luminol-enhanced chemiluminescence was used to evaluate phagocyte respiratory burst activity in both bat species (11, 33). The chemiluminescence method measures phagocyte activity by detecting light emitted during the reaction of luminol with reactive oxygen species produced in the respiratory burst of activated phagocytes. The reaction mixture contained whole blood diluted 1:50 in Hank’s balanced salt solution, luminescence probe luminol (Sigma-Aldrich Merck KGaA, Darmstadt, Germany) dissolved in borate buffer, and Zymosan A (0.25 mg/ml; Sigma-Aldrich Merck KGaA, Darmstadt, Germany) as a phagocyte activator. Chemiluminescence was recorded for two hours at two-minute intervals to obtain kinetic curves. All measurements were performed at an incubation temperature of 25 °C. Light emission was measured using a Cytation 3 M reader (BioTek Instruments, Inc., Winooski, VT, USA).Using chemiluminescence to represent phagocyte respiratory burst intensity over time, we were able to calculate the following phagocyte activity parameters (33): time to start of response (T_start_), time of peak response (T_max_), time to end of response (T_end_), peak value (peak), and total phagocyte capacity (integral). T_start_ represents the time (sec) elapsed until the chemiluminescence curve started to grow. T_max_ represents the time (sec) of the highest chemiluminescence value. T_end_ reflects the time (sec) necessary for the curve to reach its endpoint, when all phagocyte activity was exhausted. Peak in the chemiluminescence curve (measured in relative light units, RLU) reflects the maximum respiratory burst intensity. Integral (total phagocyte activity) was defined as the reaction curve area (calculated directly from the raw data by the luminometer) (for more details: 33).

We tested for interspecific difference in phagocyte activity between the active (May) and hibernation (March) seasons using the Wilcoxon-Mann-Whitney test. We expected that the deviations of immune response during winter would be best reflected at the end of deep hibernation (March), which was then compared to immune response of active individuals (May). Next, we partitioned the samples into three clusters (clustering performed separately for *M. myotis* and *N. noctula*) using the K-means clustering method based on phagocyte activity parameters (T_start_, T_max_, T_end_, peak and integral). Prior to clustering, all variables were standardized (centered to zero mean and scaled to unit variance) to ensure equal contribution of each parameter. Data was clustered to identify groups of individuals in distinct physiological states (or strength of phagocyte activity). The number of clusters (k = 3) was selected *a priori* to represent biologically meaningful grouping corresponding to null, low and high phagocyte activity states. After clustering, the data measured during the winter months for both species were pooled into three groups according to their defined clusters. One group with null phagocyte activity was excluded from further analysis, while the remaining two groups were classified as Cluster 1 (low phagocyte activity) and Cluster 2 (high phagocyte activity). We then tested the proportion of individuals with null phagocyte activity during the winter months using a two-sample Z-test for proportions. Differences in phagocyte activity between species within the same cluster were assessed using the Wilcoxon-Mann-Whitney test, while differences in phagocyte activity within species across three winter samplings were tested using the Kruskal-Wallis test. For phagocyte burst kinetics, we fitted generalized additive models (GAMs) with a Tweedie distribution and log link, using restricted maximum likelihood (REML) estimation. All statistical analyses were performed using Statistica for Windows v. 13.3 or the R software, supported by the *cluster* (34), *factoextra* (35) and *usedist* (36) and *mgcv* (37) packages.

## Results

**Figure 1:**
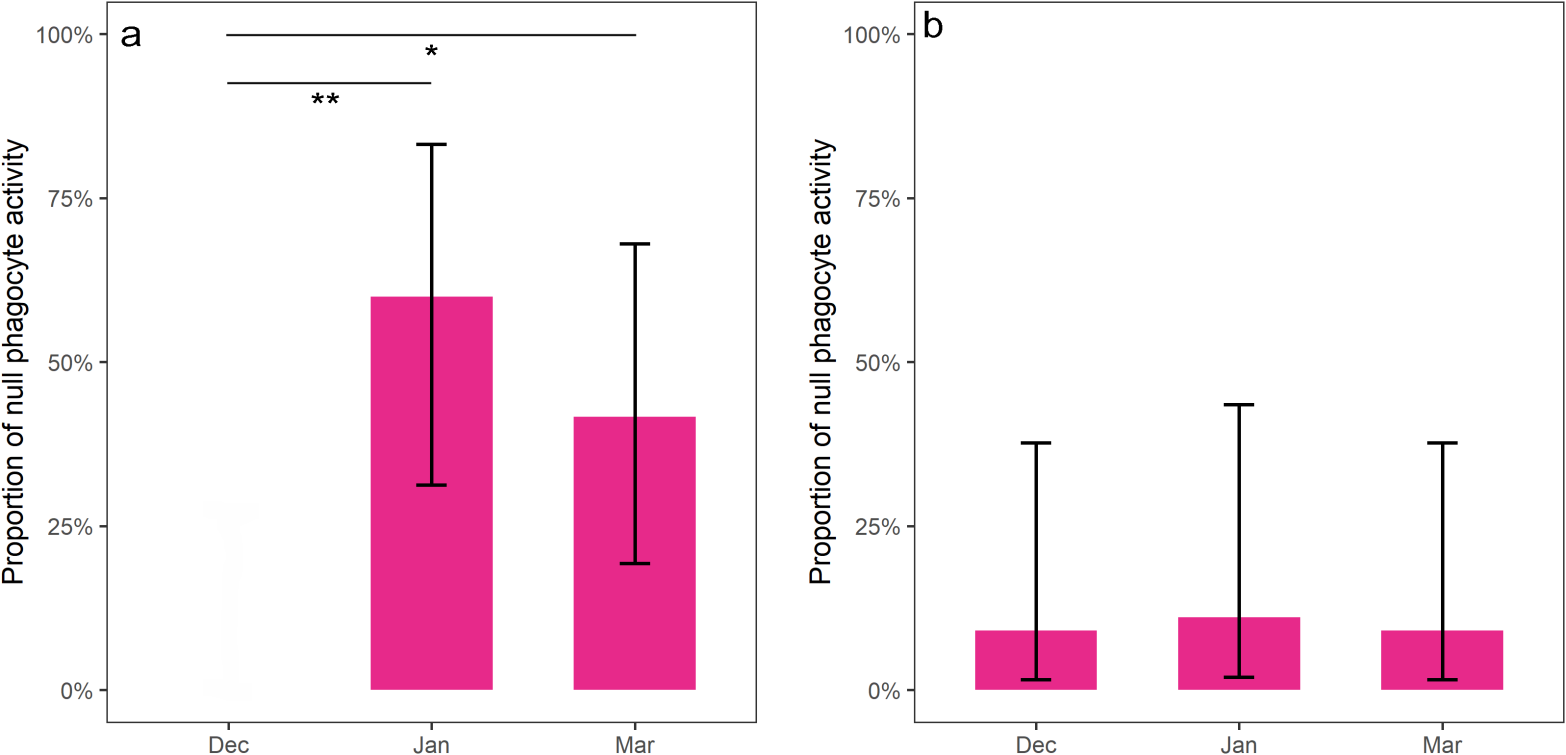
The proportion of individuals with a null phagocyte response (purple) increased in *M. myotis* during the deep hibernation period **(a)**, but it did not change in *N. noctula* throughout the hibernation **(b)**. Statistical significance *p < 0.05 and **p < 0.01, error bars represent 95% confidence intervals.

Our results demonstrate that the two bat species with different hibernation ecology exhibit distinct patterns of phagocyte respiratory burst. Unlike *N. noctula*, which showed no difference in phagocyte activity parameters (aside from T_max_ (Z = 3.184; p < 0.001)) between the active and hibernation seasons, phagocyte activity in *M. myotis* differed significantly. During the hibernation season, *M. myotis* exhibited a significantly lower peak (Z = -2.853; p = 0.004), delayed T_start_ (Z = 2.814; p = 0.005), earlier T_end_ (Z = -2.211; p = 0.027) and a lower integral (Z = -2.613; p = 0.009) compared to the active season (see **Supplementary Materials** for comparison plots of phagocyte activity parameters between active (May) and hibernation (March) season). The proportions of *N. noctula* exhibiting null phagocyte activity did not differ over the winter months (χ² = 0.3; p = 0.86). However, significantly more *M. myotis* individuals exhibited no phagocyte activity during the deep hibernation period in January (Z = -3.46; p < 0.01) and March (Z = -2.46; p < 0.05) compared to December (**Figure 1**). Meanwhile, phagocyte activity parameters did not differ significantly between hibernation months in particular bat species (**Supplementary Table 1**), except for T_start_ in *M. myotis* (H = 7.25; p = 0.027), which was significantly shorter in January compared to December. All *post-hoc* comparisons are included in **Supplementary Table 2**.

**Figure 2:**
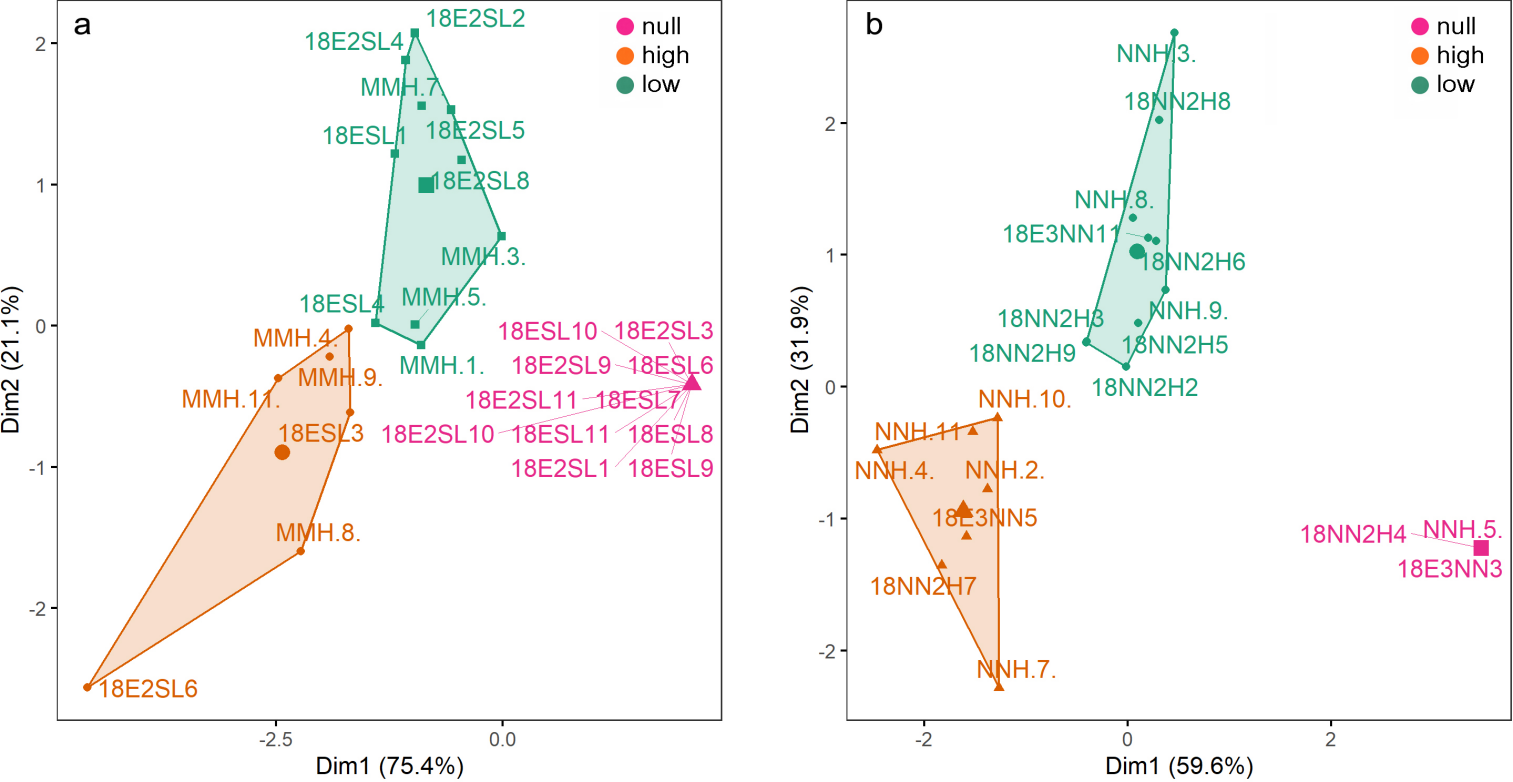
Clusters defined via K-means clustering based on phagocyte respiratory burst parameters of **(a)** *M. myotis* and **(b)** *N. noctula*. Purple clusters represent individuals with no phagocyte activity, green clusters indicate low phagocyte activity, and orange clusters high phagocyte activity.

**Figure 3:**
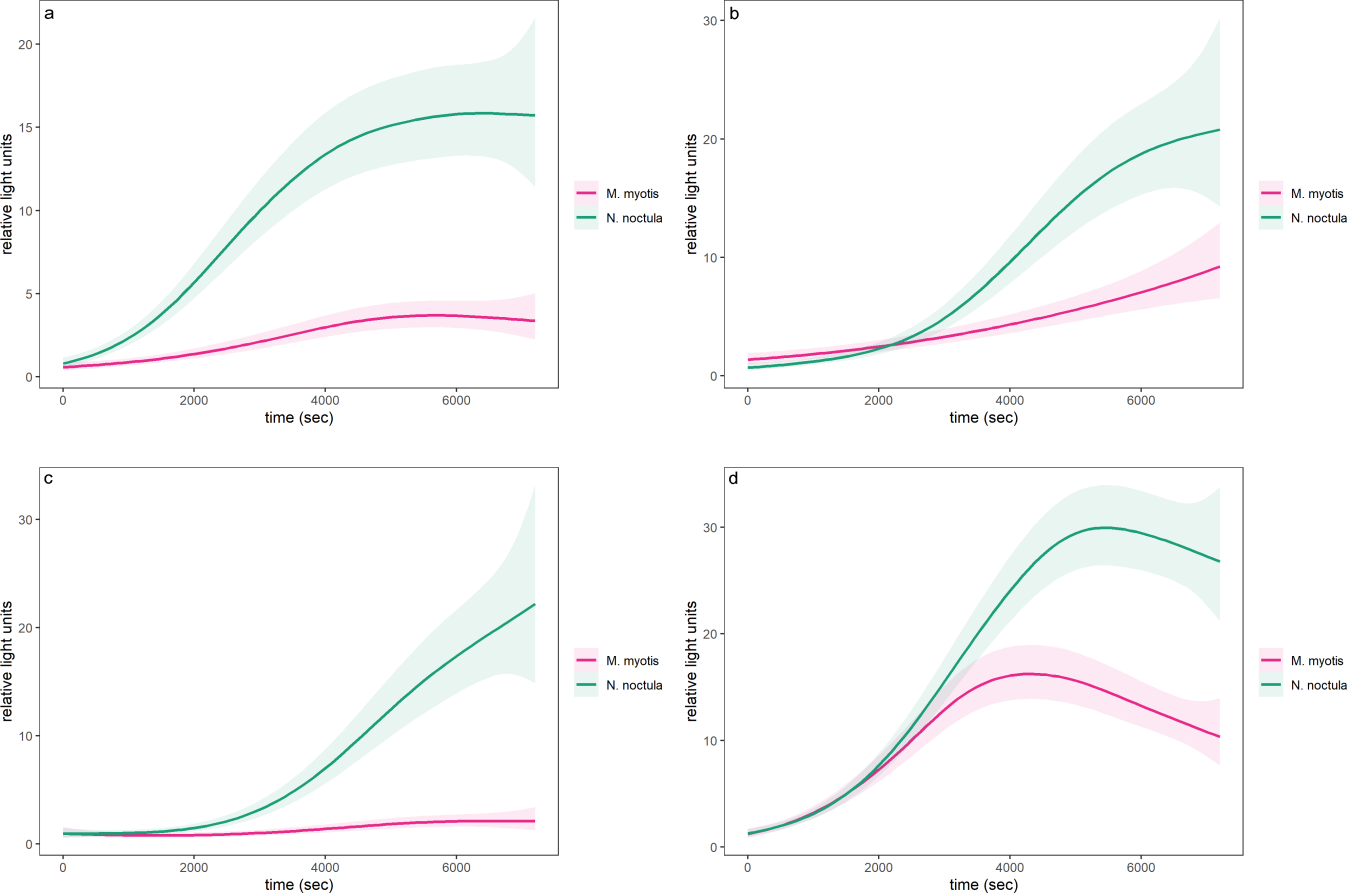
Phagocyte respiratory burst kinetics based on chemiluminescence in *M. myotis* (solid line) and *N. noctula* (dashed line) during deep hibernation [**(a)** December, **(b)** January, **(c)** March] and the active season [**(d)** May]. Solid lines represent GAM fits with a Tweedie error distribution (log link), bands represent 95% confidence intervals.

**Figure 4:**
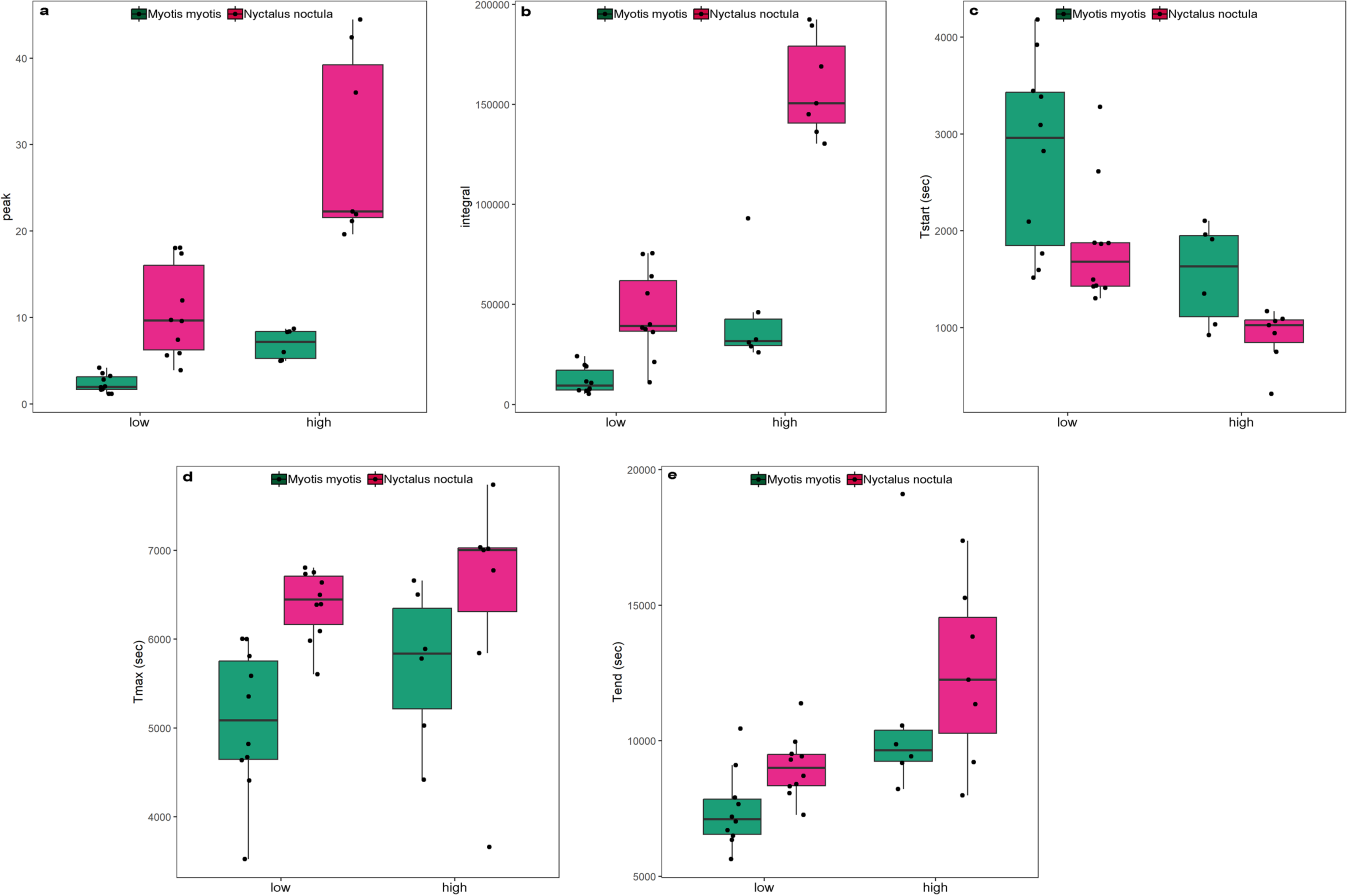
Phagocyte respiratory burst parameters **(a)** peak, **(b)** integral, **(c)** T_start_, **(d)** T_max_, **(e)** T_end_ measured during the winter months in *M. myotis* and *N. noctula* in the low phagocyte activity (low) and the high phagocyte activity group (high). Whiskers represent non-outlier data range.

For both species, K-means clustering clearly separated three clusters of individuals based on their phagocytic activity (or physiological state) (**Figure 2**). Individuals of *M. myotis* and *N. noctula* with no phagocyte activity were separated from individuals with low phagocyte activity (Euclidean distance = 2.958 and 4.064, respectively), and from those with high phagocyte activity (Euclidean distance = 4.209 and 5.104, respectively). Individuals with low phagocyte activity were separated from those with high phagocyte activity (Euclidean distance= 2.496 in *M. myotis* and 2.617 in *N. noctula*). The smooth term of time for phagocyte activity kinetics based on chemiluminescence was significant in all sampling points (December: edf = 3.75; F = 87.8; p < 0.001; January: edf = 3.66; F = 71.46; p < 0.001; March: edf = 4.53; F = 80.92; p < 0.001; May: edf = 4.89; F = 163.8; p < 0.001). Overall, phagocyte activity kinetics indicated a significant difference in response between the two species, during the hibernation (December: t = 24.41; p < 0.001, January: t = 6.8; p < 0.001; March: t = 23.17; p < 0.001; **Figures 3a–c**) and active seasons (May: t = 8.32; p < 0.001; **Figure 3d**). We found evidence that respiratory burst parameters differed between the two bat species in both the low phagocyte activity group (Cluster 1) and the high phagocyte activity group (Cluster 2), with *N. noctula* exhibiting a stronger response in both clusters with regard to quantitative respiratory burst parameters (peak and integral; **Table 1**, **Figures 4a, b**). Furthermore, *N. noctula* exhibited earlier onset of immune activity (T_start_, **Figure 4c**) and a prolonged response (T_max_, **Figure 4d**; T_end_, **Figure 4e**) compared to *M. myotis* (**Table 1**).

**Table 1:** Interspecific differences in phagocyte respiratory burst parameters in the low phagocyte activity group (cluster 1) and the high phagocyte activity group (cluster 2).

Parameter	Cluster	*Z*	*p*
peak	1	-3.704	**< 0.001**
	2	-3.000	**0.003**
integral	1	-3.402	**< 0.001**
	2	-3.000	**0.003**
T_max_	1	*-3.402*	**< 0.001**
	2	-1.714	0.086
T_start_	1	2.343	**0.019**
	2	1.857	0.063
T_end_	1	-2.570	**0.010**
	2	-0.857	0.391

Results that are significantly different are bolded.

## Discussion

Variability in the chiropteran immune system has evolved alongside bat species diversity, adapting to habitat-specific pathogen pressures and climatic challenges to maximize fitness (38–40). Here, we demonstrate contrasting functional immune responses of two European bat species throughout their hibernation. The differences observed in phagocyte respiratory burst between cave-hibernating *M. myotis* bats and above-ground hibernating *N. noctula* bats are likely related to their hibernation strategies and optimization of physiological process costs to survive the winter. As predicted, based on data showing asynchrony in bat torpor and arousal patterns to be common, we also observed differences among individuals within the species studied (41).

Hibernation behaviour is species-specific, even among bats within the same shelter (42). This difference is more pronounced among bat species that employ different types of hibernacula. Under stable cave conditions, bats can minimize their energy expenditure over the entire hibernation period, however, both innate and adaptive immune responses will be significantly suppressed (38). Our study confirmed such a relationship only in cave-dwelling hibernators after examining innate immune responses during the early and deep phases of hibernation. While phagocyte activity in hibernating *M. myotis* bats was suppressed compared to the active season, most phagocyte activity parameters in *N. noctula* bats were higher and did not differ between the hibernation and active seasons. This may be because *N. noctula* bats are subject to rapid temperature changes and show more activity in their hibernacula, or even fly outside (27, 28). From a metabolic point of view, low responsiveness to microclimatic changes would be an appropriate strategy for above-ground hibernators (43). However, active innate immunity incurs high maintenance costs (44, 45), and winter foraging may compensate for the energy losses. The relationship between physical activity, immune function and disease susceptibility is complex (46). Moderate levels of physical activity are associated with the lowest risk of disease and highest levels of immune function, while excessive physical activity can lead to down-regulation of immune defences, making animals more susceptible to disease. This trade-off occurs because the energetic costs of maintaining an innate immune system can divert resources away from immune functions during periods of high physical activity, such as reproduction and migration (47). While our knowledge of the links between physical activity and immune function in *N. noctula* bats is currently limited, our results suggest that this migratory species does not prioritise specific behaviour and movement activity in hibernacula over its phagocyte responses. Toshkova et al. (48) has shown that the diversity of antigen-binding specificities of bat IgG antibodies collapses substantially at temperatures representing inactive metabolic states, whereas elevated temperatures, typical of active flight, broadens the spectrum of recognised antigens significantly. Similarly, migrating Nathusius’ pipistrelle (*Pipistrellus nathusii*) selectively downregulate the functional role of cellular effectors, which may help save energy, apparently relying more strongly on humoral immunity responses when encountering pathogens (49).

Conversly, cave-hibernating bats have limited fat reserves and have evolved additional energy-saving strategies, such as minimal flight activity within the hibernation site (50), moving to colder areas (18), prolonging the hibernation period (17), and utilising cold arousals (41). However, immunity is energetically costly and competes with other physiological body functions for resources. Cave hibernators must therefore minimize the energy invested in phagocytosis, which explains why the proportion of specimens exhibiting no immune response increases during the hibernation period. Differential expression of plasma proteins in hibernating *M. myotis* compared to active bats has also been demonstrated, showing the modulation of proteins involved in various physiological processes, including innate immunity (10). In *M. myotis*, reactivating the generation of immune cells after the full hibernation period helps to reduce overall energy consumption. The hibernation period, however, is interrupted by arousals, during which the bats increase their body temperature, a process particularly noticeable in cave-hibernating species. Several hypotheses regarding these periodic arousals have been challenged (51). For example, it has also been suggested that periodic arousal may have evolved to reactivate the dormant immune system and clear accumulated pathogens (8, 38). This occurs in most hibernators, as extreme leukopenia and the drastically compromised functionalities of almost all immune system components are typical hibernation phenomena, both of which generally revert immediately upon arousal (4, 12). Although all leukocyte subtypes can be rapidly restored from tissues to blood upon emergence, this is not the case for all hibernating species. For example, in the edible dormouse (*Glis glis*), depleted phagocyte (neutrophils and monocytes) stores recover only slowly (52). In the case of *M. myotis*, which hibernate in caves, it has previously been demonstrated through *in vitro* experiments that phagocytic activity can be restored within minutes, in a temperature-dependent manner, and that immune cells retain some functional capacity at low temperatures (15). The best predictors of phagocyte activity in early post-emergence *M. myotis* and *N. noctula* bats are leukocyte count, haemoglobin, glucose, total dissolved carbon dioxide and chloride levels (11), reflecting the animal’s physiological state and blood metabolic and cellular characteristics.

Although the differences showed in this study are consistent with contrasting hibernation strategies of the studied species, other factors including phylogeny, morphology, foraging ecology or life-history strategies may also contribute to the observed patterns independently of hibernation ecology. Moreover, our data are limited with relatively small sample sizes per time point and the focus on only two species. Therefore, to disentangle the contribution of hibernation ecology and species-specific traits to immune response variability, future research should aim to broader comparative studies across a wider taxonomic range.

In conclusion, an understanding of seasonal variations in immune response in different bat species has important implications for conservation medicine and for developing strategies to manage bat populations in the face of emerging infectious diseases and environmental change. As bats are known to carry various zoonotic pathogens, differences in their immune responses could influence their role in infection dynamics and transmission to other wildlife and humans.

## Data Availability

The raw data supporting the conclusions of this article will be made available by the authors, without undue reservation.
